# HokUS-10 scoring system predicts the treatment outcome for sinusoidal obstruction syndrome after allogeneic hematopoietic stem cell transplantation

**DOI:** 10.1038/s41598-023-43806-3

**Published:** 2023-10-13

**Authors:** Souichi Shiratori, Kohei Okada, Junichi Sugita, Mutsumi Nishida, Takahito Iwai, Shuichi Ota, Daigo Hashimoto, Takanori Teshima

**Affiliations:** 1 Department of Hematology, Hokkaido University Hospital, N15 W7, Kita-ku, Sapporo, Hokkaido 060-8638 Japan; 2 Department of Hematology, Sapporo Hokuyu Hospital, Sapporo, Japan; 3 Diagnostic Center for Sonography, Hokkaido University Hospital, Sapporo, Japan; 4 Department of Hematology, Faculty of Medicine, Hokkaido University, Sapporo, Japan

**Keywords:** Risk factors, Medical research, Outcomes research, Haematological cancer, Imaging

## Abstract

Hepatic sinusoidal obstruction syndrome (SOS) is a severe and life-threatening complication after allogeneic hematopoietic stem cell transplantation (HSCT). We conducted a multi-center retrospective study to evaluate the utility of our ultrasonographic scoring system for the diagnosis of SOS (HokUS-10) in predicting SOS-related mortality (SOS-RM). We analyzed a total of 42 patients who developed SOS after HSCT. The cumulative incidences of SOS-RM, non-relapse mortality (NRM), and overall survival at day 180 after the diagnosis of SOS were 26.4%, 28.8% and 54.5%, respectively. The area under the receiver operating characteristic curve analysis showed that the optimal cut-off value of HokUS-10 total score to predict SOS-RM was 8 points after the treatment of SOS. In the individual HokUS-10 score, ascites and portal vein flow-related scores (PV mean velocity and PV flow direction) after the treatment of SOS were shown as significant risk factors for SOS-RM. Our study suggested that US findings after the treatment can predict the treatment outcomes for SOS.

## Introduction

Hepatic sinusoidal obstruction syndrome (SOS) is a severe and life-threatening complication after allogeneic hematopoietic stem cell transplantation (HSCT), characterized by hepatomegaly with quadrant pain, jaundice, and ascites^1^. Its risk depends on patient characteristics, disease status, conditioning, and prior history of treatments^2–4^. Severe SOS is a risk for multi-organ failure and high mortality^5^. Therefore, rapid diagnosis and early intervention are critical to improve treatment outcome against SOS. We have established an ultrasonography (US) -based scoring system for the diagnosis of SOS: the Hokkaido US-based scoring system (HokUS-10), consisting of 10 parameters including hepatomegaly, gallbladder wall thickening, ascites, portal vein (PV) and paraumbilical vein (PUV) dilatation, and abnormal blood flow signals in the PV and PUV^6^. However, the prognostic impact of US findings on treatment outcomes for SOS has never been elucidated.

## Materials and methods

### Patients and study design

We conducted a multicenter retrospective study to evaluate the association of HokUS-10 scores and treatment outcomes, including engraftment, acute graft-versus-host disease (GVHD), relapse, non-relapse mortality (NRM), overall survival (OS), and SOS-related mortality (SOS-RM) in patients with hematological malignancies who received HSCT between March 2010 and June 2021 in Hokkaido University or Sapporo Hokuyu Hospital, and were clinically diagnosed SOS as modified Seattle criteria^7^ or EBMT criteria^3^ and evaluated HokUS-10 before and after the treatment (Table S1). The cut-off score of HokUS-10 for the diagnosis of SOS was defined to 5 (maximum score, 13)^6^. The study was performed in accordance with institutional ethical guidelines, including the World Medical Association Declaration of Helsinki, and was approved by the institutional review boards of the Hokkaido University (No. 021-0096). Informed consent was obtained from each patient for participation in the study. For patients who could not obtain informed consent, we disclosed the information about this clinical study on the website of our institution and guarantee the opportunity for refusal (opt-out). Since this study is a retrospective study, opt-out disclosure eliminates the need for patient consent.

### Definitions

Neutrophil engraftment was defined as an absolute neutrophil count > 0.5 × 10^9^/L on the first of 3 consecutive days, and platelet engraftment was defined as an absolute platelet count > 2.0 × 10^10^/L without transfusion support on the first of 7 preceding days. Acute GVHD was graded according to the consensus criteria^7^. Non-relapse mortality (NRM) was defined as death due to any cause other than relapse. Relapse and causes of death were determined based on the decision of each clinician. SOS-RM was defined as death due to organ failure by SOS without relapse^8,9^. Overall survival (OS) was calculated from the day of SOS diagnosis, with patients alive at the time of last follow-up censored. NRM, relapse and SOS-RM were also calculated from the day of SOS diagnosis. HokUS-10 was evaluated in each institution, and was performed central review at Hokkaido University for this study.

### Statistical analysis

Statistical analysis was performed using Mann–Whitney *U*-test for continuous variables, Kaplan–Meier method, Log-rank test, and Cox proportional hazard model for OS, Gray’s test for engraftment, GVHD, relapse, NRM, and SOS-RM, and Fine and Gray competing risk regression model for NRM and SOS-RM. The cut-off value of HokUS-10 to predict SOS-RM was determined based on receiver operating characteristic (ROC)-curve analysis. Results were expressed as hazard ratio (HR) with the 95% confidence interval (95% CI). A value of *P* < 0.05 was used to determine statistical significance. All analyses were performed with EZR (Saitama Medical Center, Jichi Medical University, Japan), which is a graphic user interface for R (The R Foundation for Statistical Computing, Vienna, Austria)^10^.

The authors confirm that this study was conducted in accordance with relevant national, international, and institutional guidelines. The datasets used and/or analyzed during the current study available from the corresponding author on reasonable request.

## Results

### Patients and transplant characteristics

Patient and transplant characteristics in this study are shown in Table 1. A total of 42 patients were included in this study. The median patient age at the time of transplant was 50 years, ranging from 19 to 67 years. Diagnoses included acute myeloid leukemia (n = 11), malignant lymphoma (n = 11), acute lymphoblastic leukemia (n = 9), myelodysplastic syndrome (n = 6), myeloproliferative neoplasm (n = 4), and multiple myeloma (n = 1). Twenty-two patients were in complete remission at the time of transplantation. Seventeen patients received peripheral blood stem cell transplantation, 13 patients received cord blood transplantation, and 12 patients received bone marrow transplantation, respectively. The conditioning regimen was either total body irradiation-based myeloablative conditioning (n = 15), busulfan-based myeloablative conditioning (n = 13), or reduced-intensity conditioning (n = 14). Ten patients had prior history of transplantation. The median interval from HSCT to SOS diagnosis was 16 days, ranging from 4 to 133 days. Fifteen patients were diagnosed by the modified Seattle criteria, 11 patients were diagnosed by the EBMT classical criteria, and the other 16 patients were diagnosed by the EBMT late-onset criteria. EBMT severity included grade 1 (n = 8), grade 2 (n = 10), grade 3 (n = 15), and grade 4 (n = 9), respectively. Organ failure was observed in 10 patients at diagnosis, including renal failure (n = 6), respiratory failure (n = 3), and both (n = 1). Sixteen patients received defibrotide (DF) and the other received recombinant thrombomodulin (rTM) for the initial treatment.

**Table 1:** Patient characteristics.

Variable	N = 42
Median age (range)	50 (19—67)
Sex (Male / Female)	29 / 13
Disease	
Acute myeloid leukemia	11
Malignant lymphoma	11
Acute lymphoblastic leukemia	9
Myelodysplastic syndrome	6
Myeloproliferative neoplasm	4
Multiple myeloma	1
Disease status at transplantation	
Complete remission	22
Not in complete remission	20
Donor source	
Peripheral blood	17
Cord blood	13
Bone marrow	12
Conditioning	
Myeloablative conditioning	28
Total body irradiation-based	15
Busulfan-based	13
Reduced intensity conditioning	14
Prior transplantation	
0	32
1	10
Median day at diagnosis of SOS (range)	16 (4–133)
SOS diagnosis	
Modified Seattle alone	15
EBMT classical	11
EBMT late-onset	16
EBMT severity	
Grade 1	8
Grade 2	10
Grade 3	15
Grade 4	9
Organ failure	
None	32
Renal failure	6
Respiratory failure	3
Renal and respiratory failure	1
Initial treatment	
Recombinant thrombomodulin	26
Defibrotide	16

*SOS* sinusoidal obstruction syndrome.

### Clinical outcomes

**Figure 1:**
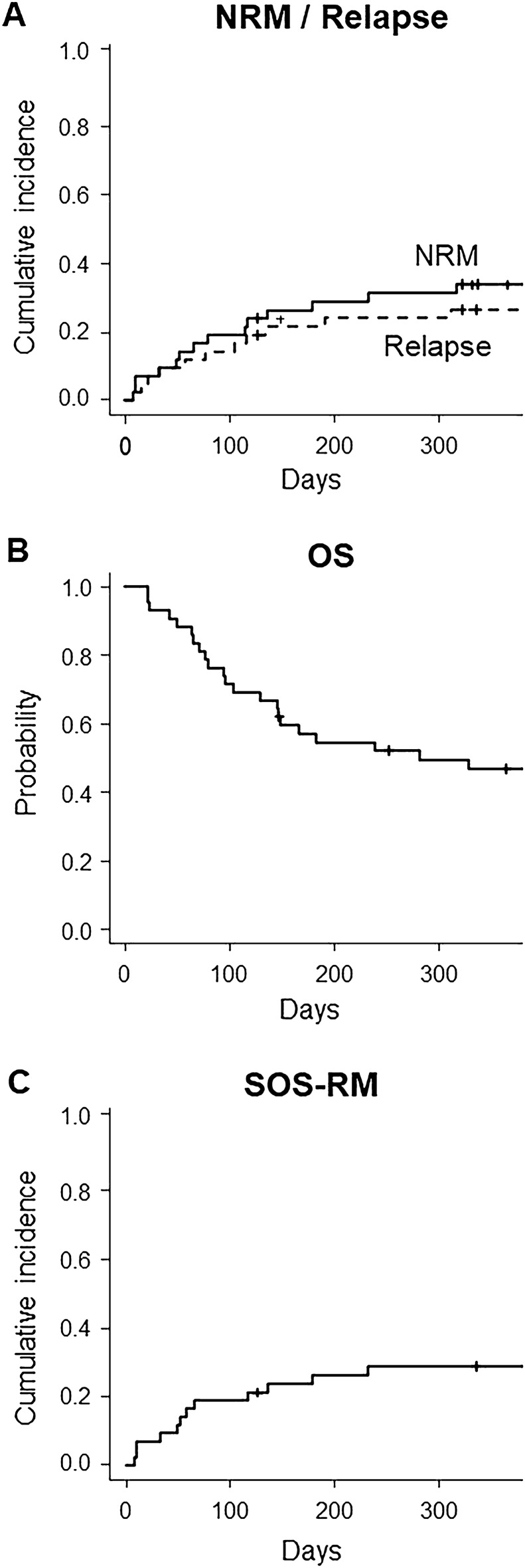
NRM/relapse, OS, and SOS-RM. The cumulative incidence of NRM (*solid lines*) and relapse (*dashed lines*) (**A**), Kaplan–Meier plots of OS (**B**), and the cumulative incidence of SOS-RM (**C**) after the start of treatment for SOS.

Neutrophil engraftment and platelet engraftment was achieved in 95.2% (95% CI 82.3–98.8%) and 58.4% (95% CI 41.5–72.0%), respectively (Fig. S1A). The cumulative incidence of overall and grade II–IV acute GVHD at day 100 were 50.4% (95% CI 34.5–64.4%) and 14.4% (95% CI 5.8–26.6%) (Fig S1B). The cumulative incidences of NRM and relapse at day 180 after the diagnosis of SOS were 28.8% (95% CI 15.9–43.0%) and 21.5% (95% CI 10.5–35.1%), respectively (Fig. 1A). With a median follow-up of 892 days for survivors, day 180 and 1-year OS after the diagnosis of SOS was 54.5% (95% CI 38.3–68.1%) and 46.8% (95% CI 31.1–61.0%), respectively (Fig. 1B). The cumulative incidences of SOS-RM at day 180 after the diagnosis of SOS were 26.4% (95% CI 14.1–40.5%) (Fig. 1C). Causes of death in 26 patients were primary diseases (n = 8), SOS (n = 11), multi-organ failure (n = 2), infection (n = 1), interstitial pneumonia (n = 1), GVHD (n = 1), and unknown cause in (n = 2) (Table S2).

### Association of HokUS-10 score with SOS-RM and transplant outcomes

**Figure 2:**
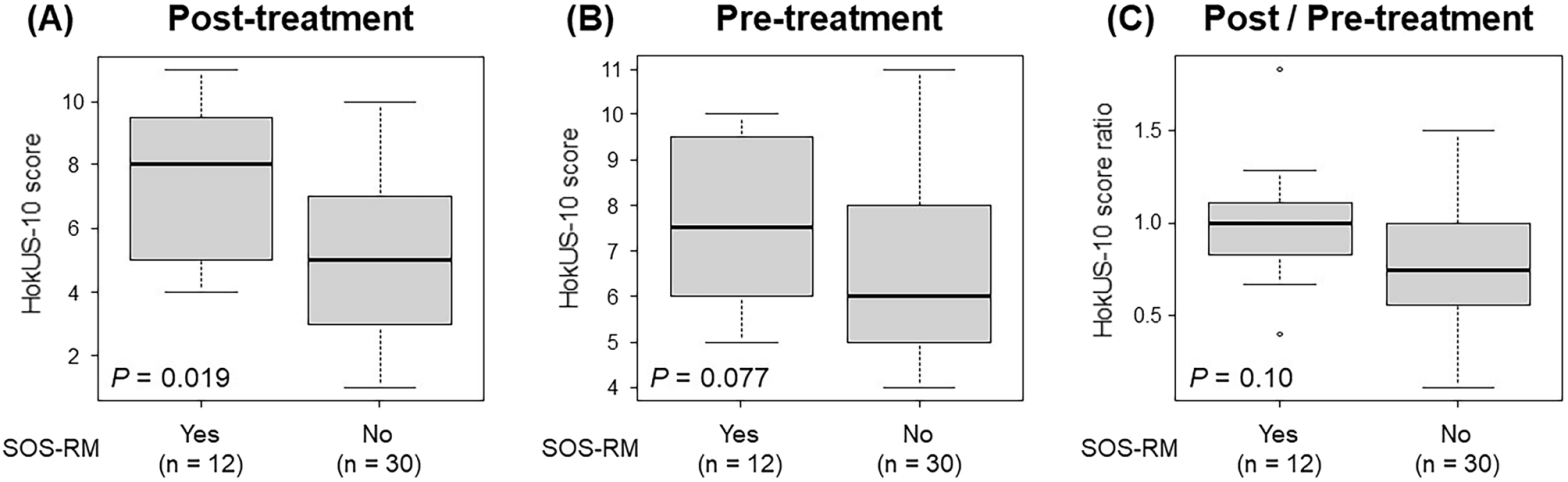
HokUS-10 total score in patients with or without SOS-RM. HokUS-10 scores in patients with or without SOS-RM post- (**A**) and pre- (**B**) treatment for SOS. Ratio of HokUS-10 scores post- and pre-treatment for SOS (**C**). The ends of the center box indicate the upper and lower quartile of the data, the line inside the rectangle indicates the median, the whiskers indicate the maximum and minimum values, and the dots outside the rectangle indicate outliers.

First, we evaluated the association of SOS-RM with total score of HokUS-10 before and after the treatment of SOS. The median days of HokUS-10 evaluation after the start of treatment was 15, ranging from 7 to 21. The median score of HokUS-10 was 6, ranging from 4 to 11, before the treatment, and 5, ranging from 1 to 11, after the treatment. Changes in an individual score of HokUS-10 before and after the treatment were shown in Fig. S2A. Most scores, except for hepatic left lobe vertical diameter and PV diameter, decreased after the treatment. The total HokUS-10 score was decreased in 25 patients, unchanged in 7 patients, and increased in 10 patients before and after the treatment. HokUS-10 scores after the treatment of SOS were significantly higher in patients with SOS-RM compared to that in patients without it (median score, 8.0 vs. 5.0, *P* = 0.019, Fig. 2A), whereas HokUS-10 scores before the treatment of SOS (median score, 7.5 vs. 6.0, *P* = 0.077, Fig. 2B) and ratio of HokUS-10 scores before and after the treatment of SOS (median ratio, 1.00 vs. 0.75, *P* = 0.10, Fig. 2C) were equivalent between the groups.

**Figure 3:**
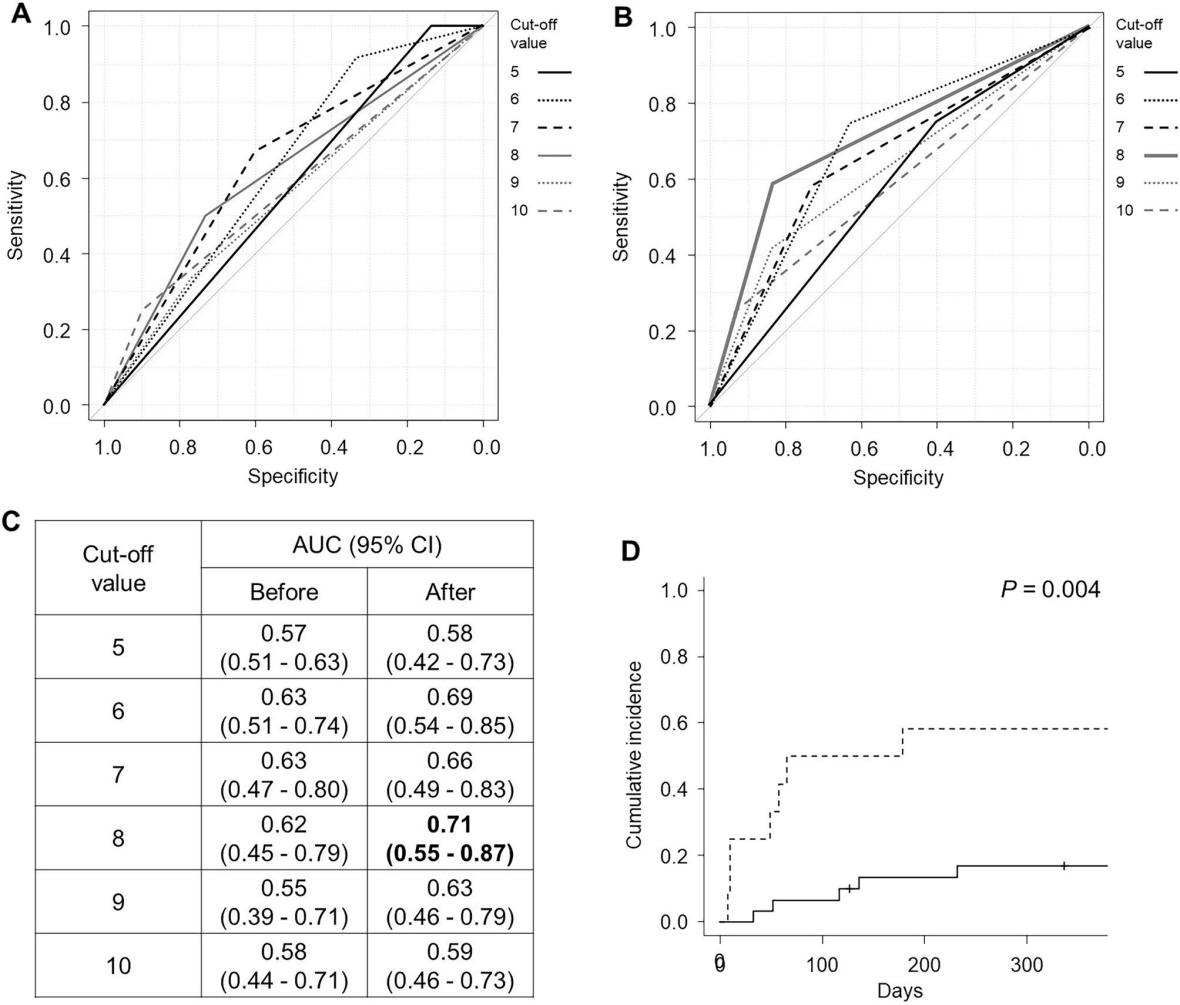
ROC curve analysis of HokUS-10 scoring system to predict SOS-RM. ROC curves to determine the cut-off values with the highest predictive performance by HokUS-10 scoring system before (**A**) and after (**B**) the treatment of SOS. Area under the ROC curves calculated by each cut-off value (**C**). The cumulative incidence of SOS-RM in patients with < 8 (*solid line,* N = 30) or ≥ 8 points (*dashed line,* N = 12) of HokUS-10 total score after the treatment of SOS (**D**).

Next, we performed further analysis for the association of HokUS-10 total score before and after the treatment of SOS with SOS-RM. While our previous study showed the diagnostic value of 5 points on HokUS-10 for SOS, the cumulative incidence of SOS-RM at day 180 after the diagnosis of SOS was not significantly difference between in patients with < 5 and ≥ 5 points of HokUS-10 total score both before and after the treatment of SOS (before: < 5 points, 0.0% vs ≥ 5 points, 29.2%, *P* = 0.20, after: < 5 points, 13.3% vs ≥ 5 points, 33.6%, *P* = 0.31). We determined the optimal cut-off value of HokUS-10 to predict SOS-RM. The area under the ROC curve analysis showed that only 8 points after the treatment of SOS became moderate accuracy to predict day 180 SOS-RM (0.71; 95% CI 0.55–0.87, Fig. 3A–C). Consequently, the cumulative incidence of SOS-RM at day 180 after the diagnosis of SOS was significantly higher in patients with ≥ 8 points than < 8 points of HokUS-10 total score after the treatment of SOS (< 8 points, 13.5% vs ≥ 8 points, 58.3%, *P* = 0.004, Fig. 3D). HokUS-10 total score after the treatment of SOS affected significant prognostic impacts for not only SOS-RM but also NRM (< 8 points, 20.1% vs ≥ 8 points, 50.0%, *P* = 0.037) and OS (< 8 points, 63.0% vs ≥ 8 points, 33.3%, *P* = 0.028) (Table S3). Multivariate analysis showed that HokUS-10 total score after the treatment of SOS was a significant risk for SOS-RM (HR, 5.029; 95% CI 1.631–15.51; *P* = 0.005). On the other hand, no significant risk factor for NRM was identified and EBMT severity was only identified as a significant risk factor for OS (HR, 3.427; 95% CI 1.044–11.26; *P* = 0.042) (Table 2).

**Table 2:** Multivariate analysis for SOS-RM, NRM, and OS at day 180 after the diagnosis of SOS.

Variable	HR (95%CI)	*P*-value
SOS-RM		
Stem cell source	0.965 (0.497–1.873)	0.920
HokUS-10 total score	5.029 (1.631–15.51)	0.005
NRM		
Stem cell source	1.049 (0.542–2.031)	0.890
Organ failure	2.361 (0.615–9.071)	0.210
HokUS-10 total score	2.714 (0.871–8.458)	0.085
OS		
EBMT severity	3.427 (1.044–11.26)	0.042
Organ failure	2.185 (0.755–6.323)	0.15
HokUS-10 total score	2.308 (0.860–6.198)	0.097

*SOS-RM* sinusoidal obstruction syndrome-related mortality, *NRM* non-relapse mortality, *OS* overall survival.

We also evaluated the association of an individual HokUS-10 score before and after the treatment of SOS with SOS-RM (Table 3). Univariate analysis identified PV mean velocity (HR, 7.94; 95% CI 1.79–35.1; *P* = 0.006) before the treatment of SOS, and ascites (HR, 3.10; 95% CI 1.33–7.23; *P* = 0.009), PV mean velocity (HR, 4.13; 95% CI 1.28–13.4; *P* = 0.018), PV flow direction (HR, 35.6; 95% CI, 8.01–158; *P* < 0.001) after the treatment of SOS as risk factors for SOS-RM.

**Table 3:** Univariate analysis of the individual HokUS-10 score for SOS-RM.

		Before treatment			After treatment	
	Factor (n)	HR (95% CI)	*P*-value	Factor (n)	HR (95% CI)	*P*-value
Hepatic left lobe vertical diameter	Score 1 (16) Score 0 (25)	2.33 (0.74–7.33)	0.15	Score 1 (16) Score 0 (25)	0.91 (0.28–2.98)	0.87
Hepatic right lobe vertical diameter	Score 1 (42) Score 0 (0)	NE	NE	Score 1 (40) Score 0 (2)	NE	NE
Gallbladder wall thickening	Score 1 (20) Score 0 (21)	0.72 (0.23–2.20)	0.56	Score 1 (10) Score 0 (31)	1.73 (0.53–5.63)	0.36
PV diameter	Score 1 (16) Score 0 (25)	0.68 (0.22–2.13)	0.51	Score 1 (18) Score 0 (24)	2.08 (0.67–6.40)	0.20
PUV diameter	Score 2 (18) Score 0 (23)	1.31 (0.74–2.31)	0.36	Score 2 (11) Score 0 (31)	2.52 (0.82–7.75)	0.11
Amount of ascites	Score 2 (26) Score 1 (14) Score 0 (2)	2.08 (0.71–6.09)	0.18	Score 2 (20) Score 1 (11) Score 0 (10)	3.10 (1.33–7.23)	0.009
PV mean velocity	Score 1 (19) Score 0 (23)	7.94 (1.79–35.1)	**0.006**	Score 1 (23) Score 0 (19)	4.13 (1.28–13.4)	0.018
PV flow direction	Score 1 (5) Score 0 (37)	3.67 (0.94–14.3)	0.061	Score 1 (3) Score 0 (39)	35.6 (8.01–158)	< 0.001
PUV flow signal	Score 2 (24) Score 0 (17)	1.17 (0.65–2.12)	0.59	Score 2 (23) Score 0 (19)	1.38 (0.78–2.46)	0.27
Hepatic artery RI	Score 1 (21) Score 0 (21)	0.68 (0.22–2.06)	0.49	Score 1 (16) Score 0 (26)	1.84 (0.61–5.54)	0.28
Total score	≥ 8 points (14) < 8 points (28)	2.40 (0.80–7.17)	0.12	≥ 8 points (12) < 8 points (30)	5.01 (1.66–15.1)	0.004

*HR* hazard ratio, *PV* portal vein, *PUV* para-umbilical vein, *RI* resistive index.

## Discussion

This multi-center retrospective study showed that HokUS-10 scores 2 weeks after the initiation of SOS treatment was associated with treatment outcomes. HokUS-10 was established based on US finding reported by Lassau et al.^11^, with the high sensitivity (100%) and specificity (95.8%)^6^. The advantage of US for the diagnosis of SOS is its ability to detect blood flow abnormalities responsible for the pathogenesis of SOS. Gallbladder wall thickening, dilatation of PUV, or appearance of PUV flow signal reflects congestion of the cystic vein returning to the PV system downstream of sinusoids. However, it remained to be elucidated whether HokUS-10 could predict treatment outcomes for SOS. In this study, we showed that high total scores (≥ 8 points) and ascites and PV flow-related scores (PV mean velocity and PV flow direction) after the treatment of SOS was significantly associated with SOS-RM. EBMT criteria also emphasizes the US findings including ascites and decrease in velocity or reversal of the portal flow as the essential ultrasound evidence of SOS^3^. Especially, PV flow is converged from the stomach, intestines, pancreas, spleen, and gallbladder, which plays an important role in transporting amino acids absorbed in intestines, hormones produced in pancreas, and decomposition products excreted from spleen to liver. Failure to improve PV flow after the treatment of SOS may reflect extensive sinusoidal obstruction, suggesting poor prognosis. Previous studies also showed the association of PV flow abnormality with poor prognosis in patients with hepatic cirrhosis^12,13^. Prediction of treatment outcomes for SOS using HokUS-10 scoring system may help to determine timing of cessation of SOS treatment.

Our study has several limitations, including a retrospective design, small sample size. The day of HokUS-10 evaluation after the treatment of SOS was not fixed. Cases with rapid progression after the diagnosis of SOS were not included, because only cases who could evaluate HokUS-10 about 2 weeks after the start of treatment were included in this study. rTM was not used outside Japan, although the previous study showed comparable therapeutic effect between rTM and DF^14,15^. Treatment outcomes were comparable between patients who received DF and rTM in this study as well.

Nevertheless, our study showed the efficacy of US findings for the prediction of treatment outcomes for SOS. Larger studies should be conducted to confirm our findingsand establish a novel scoring system using US findings for treatment outcomes of SOS.

### Supplementary Information

Supplementary Figure 1.

Supplementary Figure 2.

Supplementary Table 1.

Supplementary Table 2.

Supplementary Table 3.

Supplementary Information 6.

## Data Availability

The datasets used and/or analyzed during the current study available from the corresponding author on reasonable request.
