# Supplementary figures and images for: HokUS-10 scoring system predicts the treatment outcome for sinusoidal obstruction syndrome after allogeneic hematopoietic stem cell transplantation
Source: Sci Rep. 2023 Oct 13;13:17374. doi: 10.1038/s41598-023-43806-3 (PMC10575893; doi:10.1038/s41598-023-43806-3)

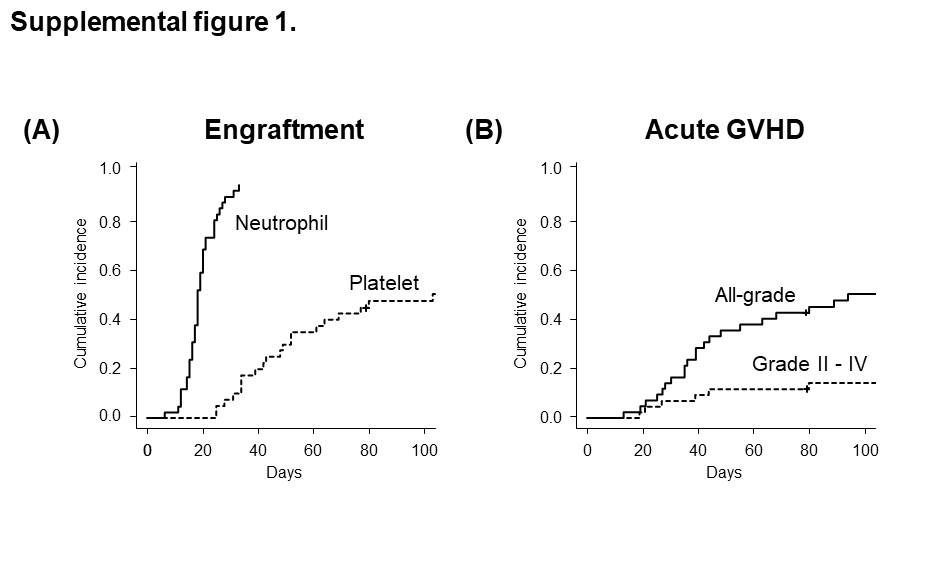

Supplement: Supplementary file 1 — Supplementary Figure 1. [file 41598_2023_43806_MOESM1_ESM.jpeg]

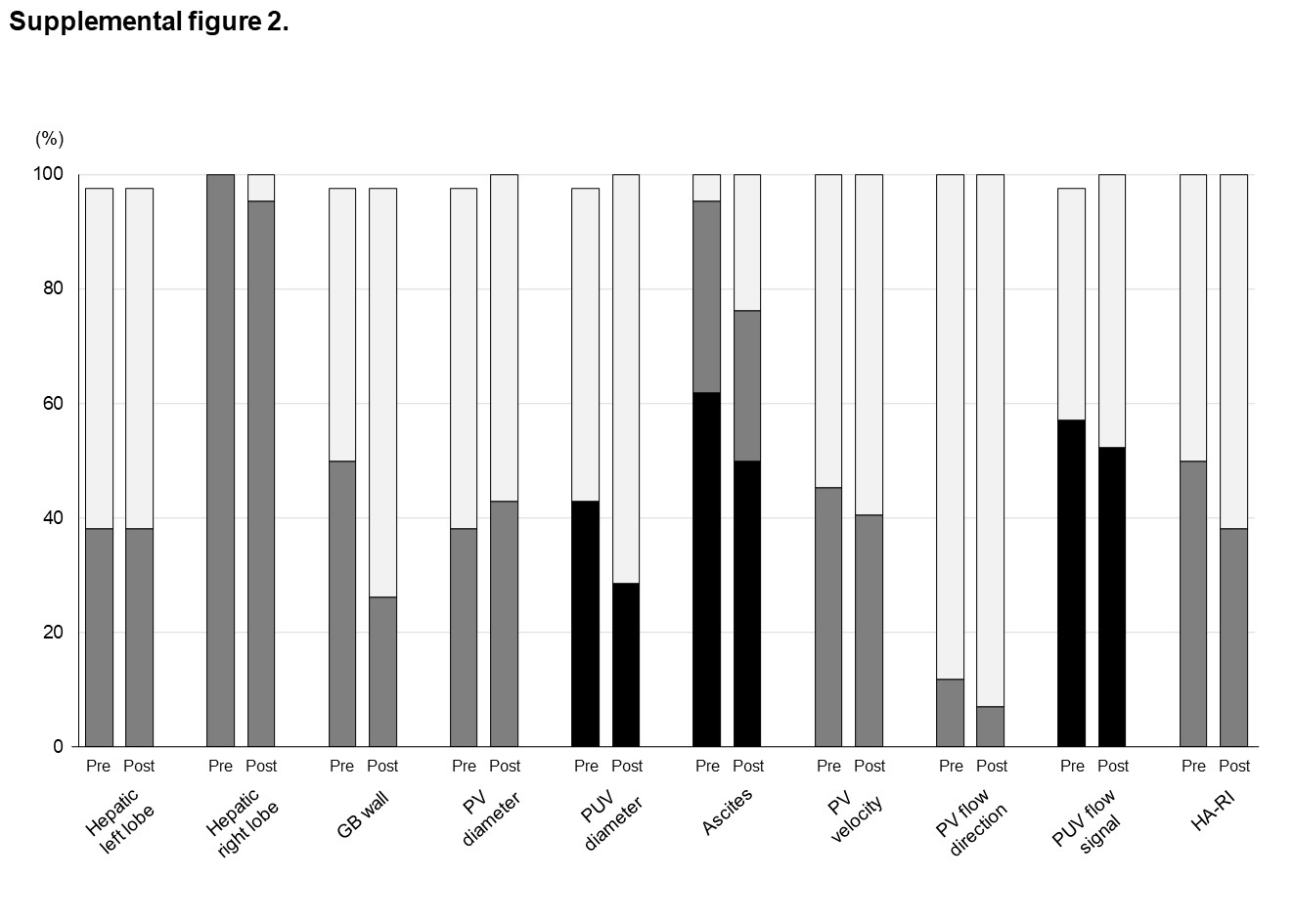

Supplement: Supplementary file 2 — Supplementary Figure 2. [file 41598_2023_43806_MOESM2_ESM.jpeg]
