# Supplementary material for: HokUS-10 scoring system predicts the treatment outcome for sinusoidal obstruction syndrome after allogeneic hematopoietic stem cell transplantation
Source: Sci Rep. 2023 Oct 13;13:17374. doi: 10.1038/s41598-023-43806-3 (PMC10575893; doi:10.1038/s41598-023-43806-3)
Supplement: Supplementary file 3 — Supplementary Table 1. [file 41598_2023_43806_MOESM3_ESM.docx]

**Supplemental table 1. HokUS-10 score**

| **Parameters** | **Description** | **Points** |
| --- | --- | --- |
| **Hepatic left lobe vertical diameter** | ≥ 70 mm | 1 |
| **Hepatic right lobe vertical diameter** | ≥ 110 mm | 1 |
| **Gallbladder wall thickening** | ≥ 6mm | 1 |
| **PV diameter** | ≥ 12 mm | 1 |
| **PUV diameter** | ≥ 2 mm | 2 |
| **Presence of ascites** | Mild | 1 |
|  | Moderate to Severe | 2 |
| **PV mean velocity** | < 10 cm/s | 1 |
| **PV blood flow direction** | Congestion or Hepatofugal | 1 |
| **Appearance of PUV blood flow signal** | Yes | 2 |
| **Hepatic artery resistive index** | ≥ 0.75 | 1 |

**Abbreviations:** PV, portal vein; PUV, paraumbilical vein.
