# Supplementary material for: HokUS-10 scoring system predicts the treatment outcome for sinusoidal obstruction syndrome after allogeneic hematopoietic stem cell transplantation
Source: Sci Rep. 2023 Oct 13;13:17374. doi: 10.1038/s41598-023-43806-3 (PMC10575893; doi:10.1038/s41598-023-43806-3)
Supplement: Supplementary file 4 — Supplementary Table 2. [file 41598_2023_43806_MOESM4_ESM.docx]

**Supplemental table 2. Causes of death**

| **Causes of death** | **N = 26** |
| --- | --- |
| **Relapse / Disease progression** | 8 (30.8%) |
| **NRM** | 18 (69.2%) |
| **SOS** | 11 |
| **Multi-organ failure** | 2 |
| **Infection** | 1 |
| **Interstitial pneumonia** | 1 |
| **Graft-versus-host disease** | 1 |
| **Unknown** | 2 |
