# Supplementary material for: HokUS-10 scoring system predicts the treatment outcome for sinusoidal obstruction syndrome after allogeneic hematopoietic stem cell transplantation
Source: Sci Rep. 2023 Oct 13;13:17374. doi: 10.1038/s41598-023-43806-3 (PMC10575893; doi:10.1038/s41598-023-43806-3)
Supplement: Supplementary file 5 — Supplementary Table 3. [file 41598_2023_43806_MOESM5_ESM.docx]

**Supplemental table 3. Univariate analysis** **for SOS-RM, NRM, and OS at day 180 after the diagnosis of SOS**

| Variables | Group | N | SOS-RM | | NRM | | OS | |
| --- | --- | --- | --- | --- | --- | --- | --- | --- |
|  |  |  | Incidence (95% CI) | *P* | Incidence (95% CI) | *P* | Incidence (95% CI) | *P* |
| Age | < 50 years | 20 | 0.35 (0.15 - 0.56) | 0.17 | 0.35 (0.15 - 0.56) | 0.29 | 0.59 (0.35 - 0.77) | 0.69 |
|  | ≥ 50 years | 22 | 0.18 (0.05 - 0.37) |  | 0.23 (0.08 - 0.42) |  | 0.50 (0.28 - 0.68) |  |
| Recipient sex | Female | 13 | 0.24 (0.05 - 0.51) | 0.78 | 0.32 (0.09 - 0.58) | 0.86 | 0.53 (0.23 - 0.76) | 0.93 |
|  | Male | 29 | 0.28 (0.13 - 0.45) |  | 0.28 (0.13 - 0.45) |  | 0.55 (0.36 - 0.71) |  |
| Disease status  at transplantation | Remission | 22 | 0.32 (0.14 - 0.52) | 0.47 | 0.36 (0.17 - 0.56) | 0.33 | 0.55 (0.32 - 0.72) | 0.92 |
|  | Not in remission | 20 | 0.20 (0.06 - 0.40) |  | 0.20 (0.06 - 0.40) |  | 0.55 (0.31 - 0.73) |  |
| Prior transplantation | 0 | 32 | 0.25 (0.12 - 0.41) | 0.84 | 0.22 (0.10 - 0.38) | 0.12 | 0.59 (0.40 - 0.74) | 0.41 |
|  | 1 | 10 | 0.30 (0.06 - 0.60) |  | 0.50 (0.16 - 0.77) |  | 0.40 (0.12 - 0.67) |  |
| Stem cell source | Bone marrow | 12 | 0.18 (0.02 - 0.45) | 0.09 | 0.18 (0.02 - 0.45) | **0.039** | 0.66 (0.32 - 0.86) | 0.21 |
|  | Peripheral blood stem cell | 17 | 0.18 (0.04 - 0.39) |  | 0.18 (0.04 - 0.39) |  | 0.59 (0.33 - 0.78) |  |
|  | Cord blood | 13 | 0.46 (0.18 - 0.71) |  | 0.54 (0.23 - 0.77) |  | 0.39 (0.14 - 0.63) |  |
| HLA | Match | 14 | 0.22 (0.05 - 0.47) | 0.62 | 0.22 (0.05 - 0.47) | 0.47 | 0.71 (0.39 - 0.88) | 0.14 |
|  | Mismatch | 28 | 0.29 (0.13 - 0.46) |  | 0.32 (0.16 - 0.50) |  | 0.46 (0.28 - 0.63) |  |
| Conditioning | Myeloablative | 28 | 0.25 (0.11 - 0.42) | 0.88 | 0.21 (0.09 - 0.38) | 0.21 | 0.64 (0.44 - 0.79) | 0.12 |
|  | Reduced-intensity | 14 | 0.29 (0.08 - 0.54) |  | 0.43 (0.16 - 0.67) |  | 0.36 (0.13 - 0.59) |  |
| SOS diagnosis 1 | Modified Seattle only | 18 | 0.28 (0.10 - 0.50) | 0.97 | 0.22 (0.06 - 0.44) | 0.37 | 0.50 (0.26 - 0.70) | 0.79 |
|  | EBMT | 24 | 0.25 (0.10 - 0.44) |  | 0.34 (0.16 - 0.53) |  | 0.58 (0.36 - 0.75) |  |
| SOS diagnosis 2 | EBMT late-onset | 16 | 0.31 (0.11 - 0.55) | 0.68 | 0.25 (0.07 - 0.48) | 0.60 | 0.44 (0.20 - 0.66) | 0.40 |
|  | Others | 26 | 0.23 (0.09 - 0.41) |  | 0.31 (0.14 - 0.49) |  | 0.61 (0.40 - 0.77) |  |
| EBMT severity | 1, 2 | 18 | 0.22 (0.07 - 0.44) | 0.48 | 0.17 (0.04 - 0.37) | 0.10 | 0.78 (0.51 - 0.91) | **0.006** |
|  | 3, 4 | 24 | 0.30 (0.13 - 0.49) |  | 0.38 (0.19 - 0.57) |  | 0.37 (0.18 - 0.56) |  |
| Organ failure | Yes | 10 | 0.40 (0.10 - 0.69) | 0.23 | 0.50 (0.15 - 0.78) | 0.059 | 0.20 (0.03 - 0.48) | **< 0.001** |
|  | No | 32 | 0.22 (0.10 - 0.38) |  | 0.22 (0.10 - 0.38) |  | 0.65 (0.46 - 0.79) |  |
| Acute GVHD before SOS | Yes | 8 | 0.38 (0.07 - 0.70) | 0.57 | 0.25 (0.03 - 0.59) | 0.65 | 0.38 (0.09 - 0.67) | 0.49 |
|  | No | 34 | 0.24 (0.11 - 0.39) |  | 0.29 (0.15 - 0.45) |  | 0.59 (0.40 - 0.73) |  |
| Initial treatment | Defibrotide | 16 | 0.19 (0.04 - 0.41) | 0.36 | 0.25 (0.07 - 0.48) | 0.62 | 0.63 (0.35 - 0.81) | 0.33 |
|  | Recombinant thrombomodulin | 26 | 0.31 (0.14 - 0.50) |  | 0.31 (0.14 - 0.50) |  | 0.50 (0.29 - 0.67) |  |
| HokUS-10 total score | < 8 points | 30 | 0.14 (0.04 - 0.28) | **0.004** | 0.20 (0.08 - 0.36) | **0.037** | 0.63 (0.43 - 0.78) | **0.028** |
|  | ≥ 8 points | 12 | 0.58 (0.27 - 0.80) |  | 0.50 (0.21 - 0.74) |  | 0.33 (0.10 - 0.59) |  |

**Abbreviations:** SOS-RM, sinusoidal obstruction syndrome-related mortality; NRM, non-relapse mortality; OS, overall survival; HLA, human leukocyte antigen; EBMT, European Society for Blood and Marrow Transplantation; GVHD, graft-versus-host disease; NA, not applicable.
