# Supplementary material for: HokUS-10 scoring system predicts the treatment outcome for sinusoidal obstruction syndrome after allogeneic hematopoietic stem cell transplantation
Source: Sci Rep. 2023 Oct 13;13:17374. doi: 10.1038/s41598-023-43806-3 (PMC10575893; doi:10.1038/s41598-023-43806-3)
Supplement: Supplementary file 6 — Supplementary Information 6. [file 41598_2023_43806_MOESM6_ESM.docx]

**Supplemental materials**

**Fig S1. Engraftment and acute GVHD**

The cumulative incidence of neutrophil (*solid lines*) and platelet (*dashed lines*) engraftment (A), and all-grade (*solid lines*) and grade II - IV (*dashed lines*) acute GVHD (B).

**Fig S2. Changed in an individual HokUS-10 score by the treatment for SOS**

Changes in an individual HokUS-10 score before and after the treatment of SOS. Light gray, dark gray, and black bars indicate score 0, 1, and 2, respectively.
